# Optimization of expression conditions for a novel NZ2114-derived antimicrobial peptide-MP1102 under the control of the GAP promoter in *Pichia pastoris* X-33

**DOI:** 10.1186/s12866-015-0389-5

**Published:** 2015-03-03

**Authors:** Ruoyu Mao, Da Teng, Xiumin Wang, Yong Zhang, Jian Jiao, Xintao Cao, Jianhua Wang

**Affiliations:** Key Laboratory of Feed Biotechnology, Ministry of Agriculture, 12 Zhongguancun Nandajie St., Haidian District Beijing, 100081 P. R. China; Gene Engineering Laboratory, Feed Research Institute, Chinese Academy of Agricultural Sciences, 12 Zhongguancun Nandajie St., Haidian District Beijing, 100081 P. R. China

**Keywords:** MP1102, GAP promoter, *Pichia pastoris*, Fermentation

## Abstract

**Background:**

The infections caused by antibiotic multidrug-resistant bacteria seriously threaten human health. To prevent and cure the infections caused by multidrug-resistant bacteria, new antimicrobial agents are required. Antimicrobial peptides are ideal therapy candidates for antibiotic-resistant pathogens. However, due to high production costs, novel methods of large-scale production are urgently needed.

**Results:**

The novel plectasin-derived antimicrobial peptide-MP1102 gene was constitutively expressed under the control of the GAP promoter. The optimum carbon source and concentration were determined, and 4% glucose (w/v) was initially selected as the best carbon source. Six media were assayed for the improved yield of recombinant MP1102 (rMP1102). The total protein and rMP1102 yield was 100.06 mg/l and 42.83 mg/l, which was accomplished via the use of medium number 1. The peptone and yeast extract from Hongrun Baoshun (HRBS, crude industrial grade, Beijing, China) more effectively improved the total protein and the yield of rMP1102 to 280.41 mg/l and 120.57 mg/l compared to 190.26 mg/l and 78.01 mg/l that resulted from Oxoid (used in the research). Furthermore, we observed that the total protein, antimicrobial activity and rMP1102 yield from the fermentation supernatant increased from 807.42 mg/l, 384,000 AU/ml, and 367.59 mg/l, respectively, in pH5.0 to 1213.64 mg/l, 153,600 AU/ml and 538.17 mg/ml, respectively in pH 6.5 in a 5-l fermenter. Accordingly, the productivity increased from 104464 AU/mg rMP1102 in pH 5.0 to a maximum of 285412 AU/mg rMP1102 in pH 6.5. Finally, the recombinant MP1102 was purified with a cation-exchange column with a yield of 376.89 mg/l, 96.8% purity, and a molecular weight of 4382.9 Da, which was consistent with its theoretical value of 4383 Da.

**Conclusions:**

It’s the highest level of antimicrobial peptides expressed in *Pichia pastoris* using GAP promoter so far. These results provide an economical method for the high-level production of rMP1102 under the control of the GAP promoter.

## Background

Antimicrobial peptides (AMPs) are widely distributed host defense molecules that are produced by certain single-cell organisms, including prokaryotic and eukaryotic organisms, as well as all multicellular plants and animals [1]. Plectasin is a recently reported novel defensin-like AMP from *Pseudoplectania nigrella* that has potent antimicrobial activity against gram-positive pathogens, such as *Staphylococcus aureus*, *Streptococcus pneumoniae* and *S. suis*, including some antibiotic-resistant strains [2,3]. Similar to vancomycin, plectasin binds to the pyrophosphate moiety of lipid II and subsequently prevents the formation of the bacterial cell wall, which inhibits colonization of the pathogens [4]. NZ2114 is a novel variant of plectasin that has significantly more potent activities than its parental peptide plectasin [5,6]. NZ2114 is active against various strains of *S. pneumoniae* and *S. aureus* in vitro and in vivo [5,7,8]. To further improve its antibacterial activity and physical and chemical properties, a new sequence named MP1102 was designed which had three mutational sites (N9E, L13V, R14K) compared to NZ2114 in our laboratory. The minimal inhibitory concentrations (MICs) of MP1102 for *S. aureus* ATCC25923, ATCC29213 and ATCC43300 were 0.021, 0.06 and 0.06 μM, respectively, and these values were equal to or more potent than those of its parental peptide NZ2114, which are 0.028, 0.11 and 0.9 μM, respectively.

The methylotrophic yeast *Pichia pastoris* has been successfully used as a host system for the expression of heterologous proteins. The high-level expression of heterologous protein by *P. pastoris* has typically been achieved using a pAOX1 expression system. However, this system is inconvenient during fermentation due to tedious processing and can create environmental pollution or fires during transportation. A constitutive glyceraldehyde-3-phosphate dehydrogenase (GAP) promoter was used as an alternative to the AOX promoter (Waterham et al. [9]). GAP is essential in carbohydrate metabolism; thus the target proteins of this promoter are expressed during the growth of the host. Consequently, cultivation is simplified because methanol is not needed as a carbon source. It gave comparable expression levels to the AOX1 promoter for some proteins, including β-Lactamase [9], xylanase [10], and human granulocyte-macrophage colony stimulating factor [11,12]. However, the operations manual of GAP promoter in *P. pastoris* indicated that toxic target proteins to the *Pichia* cell cannot be expressed with high yields (Invitrogen, Manual part no: 25–0174). Consequently, only LL37 (intracellular expression, no exact yield), human α-defensin 5 (1 mg/l) and cecropin D (485 mg/l) have been expressed with the GAP promoter [13-15]. NZ2114 is a peptide that has low toxicity to host cells and is produced at high levels in *P. pastoris* expression systems [16]. MP1102 is an NZ2114-derived antimicrobial peptide without toxicity to *P. pastoris* that has been expressed behind the AOX promoter to produce a yield of 695 mg/l (accepted by Appl Microbiol Biotechnol, DOI 10.1007/s00253-015-6394-7), which is lower than the production of NZ2114 (2,390 mg/l). The GAP promoter was used as an alternative to the AOX promoter in this study.

In the *P. pastoris* expression system using the GAP promoter, the carbon source is the key element for heterologous protein expression [17]. Additionally, the medium is another key factor for the high-level expression of heterologous protein. Various types of basal salts media (BSM) are widely used at the fermenter level in *P. pastoris* expression systems. However, the definite and limited nutrients of BSM are not suitable for the large-scale production of all proteins. An effective and cheap industrial medium is urgently needed for the industrial production of MP1102.

In the present study, the recombinant plasmid pGAPMP1102 was constructed and transformed into *P. pastoris* X-33. The optimum carbon source and its concentration were determined. Additionally, the effects of Oxoid and HRBS yeast extracts and peptones (crude industrial grades) were compared, and the effects of five different pH values were evaluated in a 5-l fermenter using fed-batch fermentation. The recombinant MP1102 (rMP1102) was purified with a cation-exchange column and confirmed by matrixassisted laser desorption/ionization-time of flight mass spectrometry (MALDI-TOF MS).

## Results

### Vector construction and screening of positive transformant

**Figure 1:**
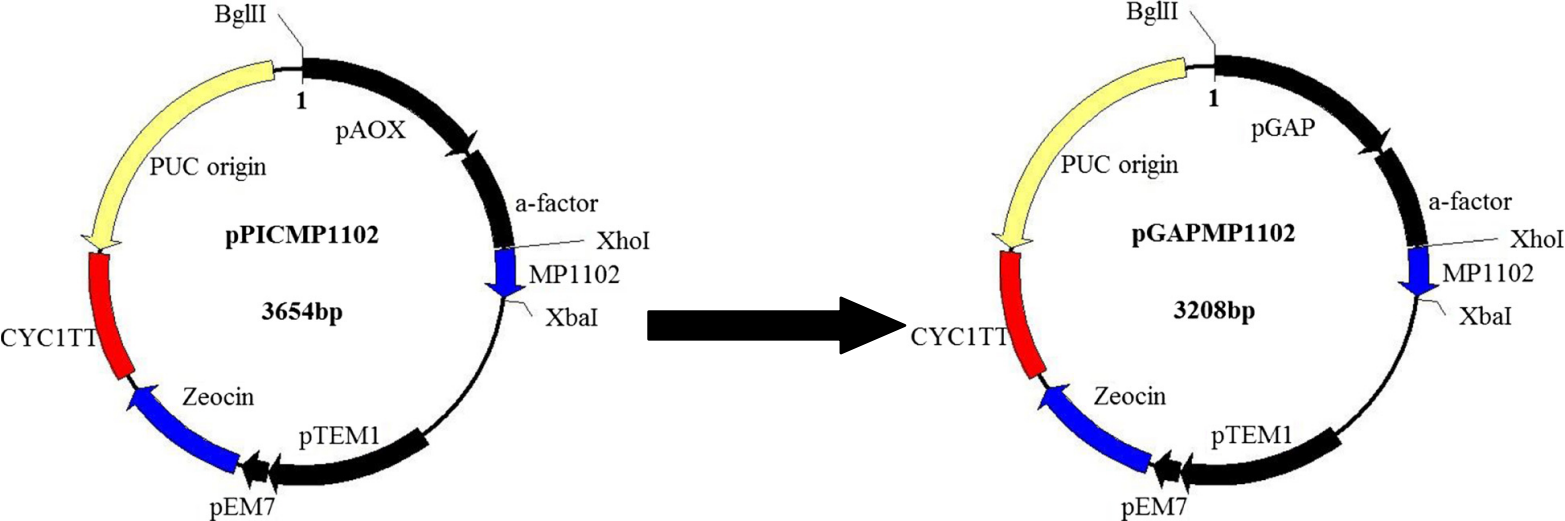
**Construction of the recombinant expression vector pGAPMP1102.** α-factor: native *S. cerevisiae* α-mating factor secretion signal, which can be self-cleaved by the endogenous Kex2 protease to leave a native sequence of target peptide; pAOX: methanol-inducible alcohol oxidase one promoter from *P. pastoris*; pGAP: glyceraldehyde-3-phosphate dehydrogenase promoter from *P. pastoris*; PTEF1: transcription elongation factor one gene promoter; PEM7: synthetic prokaryotic promoter; Zeocin: Zeocin resistance gene; pUC ori: replication and maintenance of the plasmid in *E. coli*; CYC1 TT: transcription termination region.

As shown in Figure 1, the pGAPMP1102 plasmid contained an inserted target MP1102 fragment of 120 bp. All positive transformants were verified by sequencing. The correct pGAPMP1102 recombinant plasmid was linearized with *Avr*II and transformed into *P. pastoris* X-33 by electroporation. The positive transformants were screened by PCR using GAP gene-specific and MP1102 gene-specific primers, and an empty pPICZαA vector transformant was used as the negative control (data not shown).

### Confirmation of the anti-*S. aureus* activity of the transformant and carbon source screening

**Figure 2:**
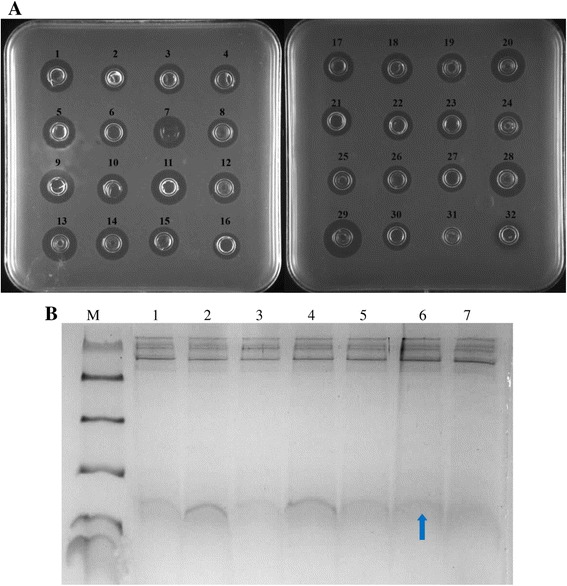
**Screening and analyses of the positive transformants. A**: Antimicrobial activity screening of the positive transformants of *Pichia pastoris* GAPMP1102. 1–30: the fermentation supernatants of the different transformants of *Pichia pastoris* GAPMP1102; 31: the fermentation supernatant of *Pichia pastoris* X-33; 32: 0.5 μg ampicillin. **B**: Tricine-SDS-PAGE analyses of the different transformants with positive antimicrobial activities. M: A total of 5 μl of protein molecular weight marker (from top to bottom: 40, 25, 15, 10, 4.6 and 1.7 kDa); Lanes 1–7: Totals of 10 μl fermentation supernatants of different transformants of *Pichia pastoris* GAPMP1102. The arrow indicates rMP1102.

Thirty positive transformants were used for fermentation in shake flasks. All transformants exhibited some level of antimicrobial activity (Figure 2A). A predominant band between 3.3 and 5.8 kDa was observed in lanes 1–7, the total protein and rMP1102 yield of up to 43.6 mg/l and 18.31 mg/l were observed after 72 h of cultivation (Figure 2B).

**Figure 3:**
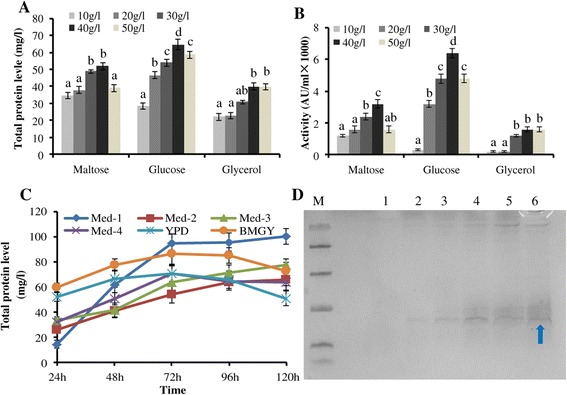
**Carbon sources and medium selection for the high-level production of rMP1102 at the shake flask level. A**, **B**: Effects of different carbon sources on the total protein levels and antimicrobial activities of the fermentation supernatants of *Pichia pastoris* GAPMP1102; **C**: Total protein levels of the fermentation supernatants of *Pichia pastoris* GAPMP1102 in the different media; **D** Tricine-SDS-PAGE analysis of fermentation supernatant of *Pichia pastoris* GAPMP1102 in Med-1. Lane M: a total of 5 μl of protein molecular weight marker (from top to bottom: 40, 25, 15, 10, 4.6 and 1.7 kDa). Lanes 1–6: 10 μl of rMP1102 fermentation supernatants taken at 0, 24, 48, 72, 96, and 120 h of induction, respectively. The arrow indicates rMP1102. Each data point is the average of three replicates, and the error bars represent the standard deviation. Different lowercase letters (a, ab, b, c) above the bar indicate significant differences between groups (P < 0.05).

Unlike the AOX1 promoter, which is inactive on glucose and glycerol and requires methanol to initiate target gene expression, the GAP promoter constitutively expresses target proteins on different carbon sources [18]. The yield of rMP1102 increased with increasing carbon source concentrations until 40 g/l. The maximum total protein level, rMP1102 yield and antimicrobial activity were 67.8 mg/l, 27.8 mg/l and 6400 AU/ml, respectively, after 96 h of cultivation at an initial glucose concentration of 40 g/l (Figure 3A, B). Additionally, rMP1102 was not secreted into the medium at high levels when low concentrations (10 and 20 g/l) of any of the three carbon sources were used. However, the yields of rMP1102 decreased to 26.16 and 18.71 mg/l in glucose and maltose, respectively at concentrations of 50 g/l (P < 0.05), which suggested that excessive high carbon source concentrations did not benefit the production of rMP1102.

### Medium selection for the high-level production of rMP1102

**Figure 4:**
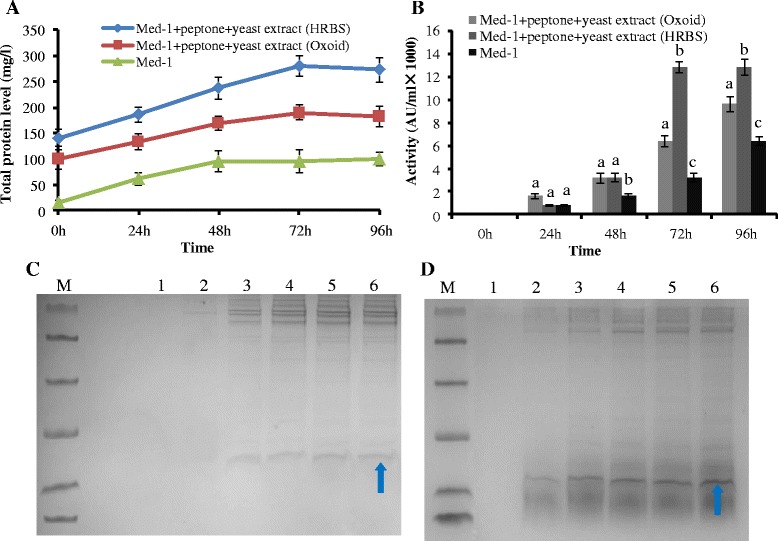
**Effects of peptone and yeast extracts from Oxoid and HRBS (crude industry level) on the production of rMP1102. A**, **B**: Effects of the peptone and yeast extracts on the total protein levels and antimicrobial activities of the fermentation supernatants of *Pichia pastoris* GAPMP1102. **C**: Tricine-SDS-PAGE analyses of the fermentation supernatants from *Pichia pastoris* GAPMP1102 in Med-1 supplemented with peptone and yeast extract from Oxoid. Lane M: a total of 5 μl of protein molecular weight marker (from top to bottom: 40, 25, 15, 10, 4.6 and 1.7 kDa). Lanes 1–6: 10 μl of the rMP1102 fermentation supernatants taken at 0, 24, 48, 72, 96, and 120 h of induction, respectively. **D**: Tricine-SDS-PAGE analysis of the fermentation supernatants from the *Pichia pastoris* GAPMP1102 in Med-1 supplemented with peptone and yeast extract from HRBS (crude industry level). Lane M: a total of 5 μl of protein molecular weight marker (from top to bottom: 40, 25, 15, 10, 4.6 and 1.7 kDa). Lane 1–6: 10 μl of rMP1102 fermentation supernatants taken at 0, 24, 48, 72, 96, and 120 h of induction, respectively. The arrow indicates rMP1102. Each data point is the average of three replicates, and the error bars represent the standard deviation. Different lowercase letters (a, b, c) above the bar indicate significant differences between groups (P < 0.05).

To identify the best medium for the high-level production of rMP1102, six different media were examined. As shown in Figure 3C, the highest total protein and rMP1102 yield of 100.06 mg/l and 42.83 mg/l were observed with Med-1 following 120 h of cultivation. To further improve this yield and identify a suitable medium for rMP1102 production, yeast extracts and peptones from Oxoid and HRBS (crude industrial grades) were added. The higher total protein, rMP1102 yield and antimicrobial activity of 280.41 mg/l, 120.57 mg/l and 12800 AU/ml were observed following the addition of industrial yeast extract and peptone to Med-1 (Figure 4A, B); this yield was 2.80 times that observed with Med-1 alone. Additionally, the total protein, rMP1102 yield and antimicrobial activity were 190.26 mg/l, 78.01 mg/l and 9600 AU/ml following the use of Med-1 with added Oxoid yeast extract and peptone (Figure 4A, B). Tricine-SDS–PAGE revealed a target band of rMP1102 between 4.4 and 10.0 kDa that was more obvious following the use of Med-1 with HRBS yeast extract and peptone than the medium that contained Oxoid (Figure 4C, D), which suggesting that industrial yeast extract and peptone were more suitable for the production of rMP1102.

### Development of fed-batch cultivation in bioreactors

**Figure 5:**
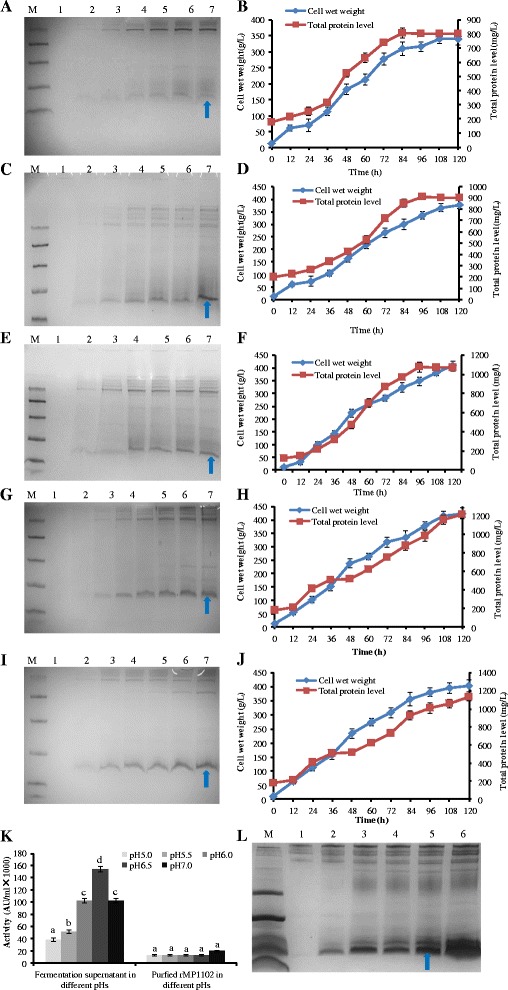
**Effects of different pHs on the yield of rMP1102 via fed-batch fermentation. A**, **C**, **E**, **G**, **I**: Tricine-SDS-PAGE analyses of the fermentation supernatants of *P. pastoris* GAPMP1102 in pHs of 5.0, 5.5, 6.0, 6.5 and 7.0, respectively. Lane M: a total of 5 μl of protein molecular weight marker (from top to bottom: 40, 25, 15, 10, 4.6 and 1.7 kDa). Lanes 1–7: Supernatants (10 μl each) harvested at 0, 24, 48, 72, 96, 108 and 120 h of cultivation, respectively. The arrow indicates rMP1102. **B, D, F, H, J**: Cell wet weights and total protein levels of the fermentation supernatants of *P. pastoris* GAPMP1102 in pHs of 5.0, 5.5, 6.0, 6.5 and 7.0, respectively. **K**: Antimicrobial activity of the fermentation supernatants of *P. pastoris* GAPMP1102 in pHs of 5.0, 5.5, 6.0, 6.5 and 7.0, respectively. **L**: Tricine-SDS-PAGE analysis of the fermentation supernatant of *P. pastoris* AOXMP1102 in pH 5.0. Lane M: a total of 5 μl of protein molecular weight marker (from top to bottom: 40, 25, 15, 10, 4.6 and 1.7 kDa). Lanes 1–6: supernatants (10 μl each) harvested at 0, 24, 48, 72, 96, and 120 h of cultivation, respectively. The arrow indicates rMP1102. Each data point is the average of three replicates, and the error bars represent the standard deviation. Different lowercase letters (a, b, c, d) above the bar indicate significant differences between groups (P < 0.05).

The experiments involving the fermentation of rMP1102 in 5-l fermenters focused on pH because pH is the key factor that influences the yields of target proteins [19]. The total protein level, rMP1102 yield and antimicrobial activity of the fermentation supernatant increased from 807.42 mg/l, 367.59 mg/l and 38,400 AU/ml, respectively, at pH 5.0 to the maximum values of 1213.64 mg/l, 538.17 mg/l and 153,600 AU/ml, respectively, at pH6.5 (Table 1). However, the corresponding values decreased to 1127.38 mg/l, 503.92 mg/l and 102,400 AU/ml at pH 7.0, which was not the perfect condition for the growth of the host and target peptide production in *P. pastoris*. This pattern of productivity paralleled the absolute antimicrobial activity values. The lowest productivity was 104464 AU/mg rMP1102, which was observed at the pH of 5.0, and the maximum max value occurred in pH 6.5 and was 285412 AU/mg rMP1102. However, productivity did not increase when the pH was raised to 7.0 (Table 1). Moreover, a wider target band resulted from pH 6.5 compared to the other pH values (Figure 5A, C, E, G, I). Additionally, the cell wet weights and total protein levels increased with the process of cultivation in all pH values (Figure 5B, D, F, H, J). The total protein level increased to the maximums at 84 and 96 h in the pHs of 5.0, 5.5, and 6.0. However, the highest total protein level and antimicrobial activity occurred at 120 h at the pHs of 6.5 and 7.0, which suggested that these conditions were beneficial for the continuous accumulation of rMP1102, whereas the target peptides accumulated faster in the low 5.0, 5.5, and 6.0 pH conditions (Figure 5B, D, F, H, J). rMP1102 was also expressed in *P. pastoris* via the inducible method at the 5-l fermenter level. The maximum total protein level was 695 mg/l at 120 h (accepted by Appl Microbiol Biotechnol, DOI 10.1007/s00253-015-6394-7), which was more than 70% reduced compared to the constitutive expression at pH 6.5. Furthermore, another non-target band appeared above rMP1102 between 3.3 and 5.8 kDa. This band might have represented incomplete cleavage by Kex2 due to the large amount of target peptides that were induced by methanol in a short time [20]. In contrast, there only the target rMP1102 was expressed in each of the assays that used the GAP promoter (Figure 5A, C, E, G, I). Very few or no other bands appeared around rMP1102, which was convenient for the process of purification.

**Table 1:** **Productivity related parameters of** ***Pichia pastoris*** **GAPMP1102 for fed-batch fermentation under different pH conditions**

	**pH 5.0**	**pH 5.5**	**pH 6.0**	**pH 6.5**	**pH 7.0**
Total protein (mg/l)	807.42 ± 26.35	913.28 ± 22.94	1076.99 ± 35.86	1213.64 ± 42.68	1127.38 ± 37.62
rMP1102 (mg/l)	367.59 ± 13.51	423.62 ± 18.27	486.35 ± 17.96	538.17 ± 21.32	503.92 ± 19.85
Activity (AU/ml × 10000)	3.84 ± 0.31	5.12 ± 0.28	10.24 ± 0.36	15.36 ± 0.43	10.24 ± 0.37
Max cell wet weight (g/l)	341.2 ± 19.41	378.5 ± 17.13	410.7 ± 16.29	424.9 ± 25.81	402.6 ± 21.74
Specific cell growth rate (*μ*)	0.188	0.189	0.208	0.224	0.198
Productivity (AU/mg rMP1102)	104464	120863	210548	285412	203207

### Purification and identification of rMP1102

**Figure 6:**
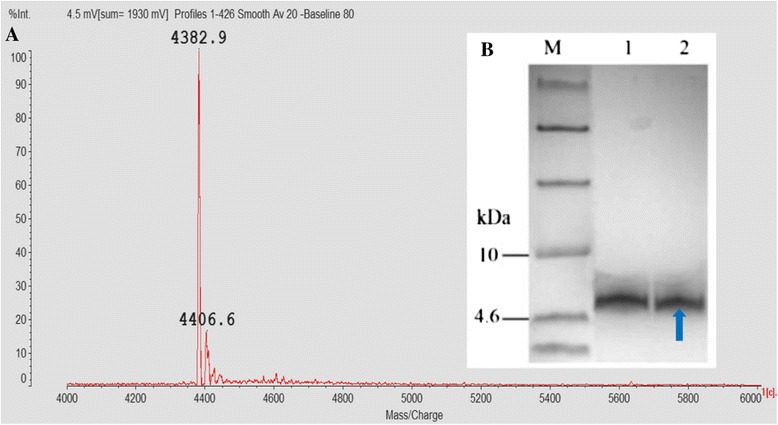
**Purification and identification of rMP1102. A**: MALDI-TOF MS analysis of the purified rMP1102. **B**: Tricine-SDS-PAGE analysis of the purified rMP1102. Lane M: A total of 5 μl of protein molecular weight marker (from top to bottom: 40, 25, 15, 10, 4.6 and 1.7 kDa). Lanes 1–2: purified rMP1102 (0.1 μg). The arrow indicates rMP1102.

rMP1102 was purified from the culture supernatant using a two-step purification protocol in a 5-l fermenter using Med-1 supplemented with industrial yeast extract and peptone at pH 6.5. As shown in Table 2, after the two-step purification, a total of 1.273 × 10^8^ AU and 376.89 mg of protein was obtained from 1000 ml of culture supernatant (Table 2). The final recovery yield, based on total activity, was 82.9%. A single band of rMP1102 from the purified sample was observed and exhibited a purity of 96.8% (Figure 6A). The results of MALDI-TOF MS of the purified rMP1102 indicated that the molecular weight of the target band was 4382.9 Da, which is consistent with its theoretical value of 4383 Da (Figure 6B).

**Table 2:** **Purification and yield of rMP1102**

	**Volume (ml)**	**Protein concentration (mg/l)**	Specific activity (AU *10000/ml)	Total activity (AU*100000)	**Recovery (%)**
Culture supernatant	1000	1213.64 ± 42.68	15.36 ± 0.43	1536	100
Sephadex G-25	2600	357.31 ± 17.14	5.37 ± 0.08	1397	90.95
SP FF					
Sepharose	820	376.89 ± 15.83	15.52 ± 0.36	1273	82.93

## Discussion

The main hosts used for AMP production are bacteria and yeasts, which are used to produce 97.4% of the heterologously expressed AMPs [21]. Due to the mechanisms of membrane disruption and cell wall compound blocking, which induce death [4,22], very low yields of target peptides are obtained by directly expressing AMPs in prokaryotic expression systems. Therefore, fusion partners, such as thioredoxin (Trx), glutathione-S-transferase (GST) and small ubiquitin-related modifier (SUMO), have been introduced to avoid host toxicity [23-25]. However, it is quite difficult to recover large fusion proteins at reasonable ratios, so this process is uneconomical and laborious. *P. pastoris* is a high-yield host for the antimicrobial peptide production and has some advantages over prokaryotic expression systems [26]. Moreover, compared to mammalian cell cultures, *P. pastoris* is more cost-effective and grows faster. Consequently, the majority of heterologous AMPs produced in yeast are expressed in *P. pastoris* [22].

The most commonly used vectors harbor the AOX promoter in *P. pastoris*, which is induced by methanol. Many AMPs have been expressed using this promoter, including hPAB-β [27], ABP-CM4 [28] and Ch-penaeidin [29]. Additionally, fungal defensin-plectasin and its derived peptides NZ2114, LHP7, and AgPlectasin have also been successfully expressed using this system in our laboratory with high production rates of 2390 mg/l, 906 mg/l, and 1285 mg/l, respectively [3,16,30,31]. However, methanol is a substance that is prone to cause fires during transportation and storage. The GAP promoter is an attractive alternative to the AOX promoter and might reduce costs and risks during the processes of fermentation and transportation. It has been shown that the various proteins, such as bacterial β-lactamase [9], D-amino acid oxidase and [32], human angiostatin [33], and functional mammalian membrane transport protein production [34], are more effectively expressed in constitutive systems than in the methanol-inducible system. In this work, all of the positive transformants exhibited some level of antimicrobial activity different levels (Figure 2A, B), However, the maximum total protein and rMP1102 yield were 43.6 mg/l and 18.31 mg/l after 72 h of cultivation (Figure 2C), which leaves much room for improvements via the optimization of fermentation.

Glyceraldehyde-3-phosphate dehydrogenase is the crucial enzyme in sugar metabolism. Consequently, the carbon source is one of the most important factors in the efficiency of the GAP promoter [9]. The maximal transport activities of rPEPT2 growing on glucose are approximately 2 and 8 times greater than those on glycerol and methanol, respectively [34]. Additionally, glucose at 40 g/l is the best choice for both cell growth and lipase production [35]. However, glycerol is a better carbon source than glucose or methanol for the production of human angiostatin [33]. It was also found that the methanol could also initiate the expression the reporter of β-lactamase, its activity was 36.36% compared to that of glucose as carbon source [9]. Therefore, three carbon sources were tested at different concentrations in our work. The findings revealed that the maximum productions in terms of total protein, rMP1102 yield and antimicrobial activity were 67.8 mg/l, 27.8 mg/l and 6400 AU/ml following 96 h of cultivation at an initial glucose concentration of 40 g/l (Figure 3A, B).

Basal salt medium (BSM) and FM22 are the defined media commonly used in the large-scale production of heterologous proteins [36]. The present work revealed that the maximum total protein level and rMP1102 yield in the defined medium were 100.06 mg/l and 42.83 mg/l, which was achieved using Med-1 (Figure 3C) at the shake flask level. Additionally, rich media might be more favorable than defined media for the production of some proteins. Yeast extract, peptone, and casamino acids are the complex media that are typically added to defined medium [36]. These media can enhance the yield of target protein and protect host cells from proteolytic degradation. It has been shown that recombinant human consensus IFN-α (IFN-Con1) mutant can be highly expressed with a maximum yield of 1.23 g/l via the addition of yeast extract and peptone [37]. Similarly, the highest yield of total protein, rMP1102 and antimicrobial activity of 280.41 mg/l, 120.57 and 12800 AU/ml was observed following the use of Med-1 supplemented with crude industrial yeast extract and peptone (Figure 4A, B). However, the yield of total protein, rMP1102 and antimicrobial activity reduced to 190.26 mg/l, 78.01 mg/l and 9600 AU/ml when Med-1 supplemented with Oxoid yeast extract and peptone was used (Figure 4A, B), which suggests that industrial media are more suitable for the high-level production of this target peptide. This difference might be due to the extensive machining processes that might keep the certain micronutrients in the industrial grade medium that improved target peptide yield. However, the exact mechanism will be studied in our future work. Moreover, the costs of industrial yeast extract and peptone were $ 9.68 and 11.29 per kilogram (Table 3), which were 3.17 and 7.57 times lower than the costs of the Oxoid components, respectively (Table 3); thus, the industrial yeast extract and peptone are likely more suitable for large-scale production.

**Table 3:** **Costs of peptone and yeast extracts**

**Medium**	**Price ($)**
Yeast extract (HRB^a^)	9.68
Yeast extract (Oxoid)	40.32
Peptone (HRB)	11.29
Peptone (Oxoid)	96.77

^a^The media from Hongrunbaoshun Co., Lod.

Because *P. pastoris* grows in a wide range of pHs, the optimization of fermentation is typically adapted within these limits to provide the best conditions for individual proteins [19]. The expression level of sEDIII-2 is highest at a pH of 5.8, and this level is 20–25% higher than those achieved at other pH values [38]. Moreover, it has been found that an induction pH of 6.8 results in a merozoite surface protein 3 (MSP3) yield of at 434 mg/L, whereas this is no product at pH 5.0 despite though cell growth being identical across all pH levels [39]. Consequently, pH values of 5.0 to 7.0 were selected for rMP1102 production at the 5-l fermenter level. The greatest amount of total protein, rMP1102 yield and antimicrobial activity were 1213.64 mg/l, 538.17 mg/l and 153,600 AU/ml in pH 6.5, with the highest productivity and specific cell growth rate of 285412 AU/mg rMP1102 and 0.224, respectively (Table 1), which corroborates the notion that, under the GAP promoter, the target peptide production was accompanied by the growth of host cells [9]. Furthermore, this was the highest yield of antimicrobial peptide achieved using the GAP promoter and thus provides a practical system for the large-scale production of antimicrobial peptides with structures similar to that of MP1102.

## Conclusions

For the first time, the novel NZ2114-derived peptide MP1102 was expressed in *P. pastoris* using the GAP promoter. Four percent initial glucose and Med-1 were identified as the best carbon source and medium, respectively, among three carbon sources and six media. Moreover, the replacement of Oxoid peptone and yeast extract (used in the research) with HRBS (crude industrial grade) was beneficial in terms of a higher yield of rMP1102. Additionally, the total protein level, rMP1102 yield, and antimicrobial activity increased from 807.42 mg/l, 367.59 mg/l, and 38,400 AU/ml, respectively, in pH 5.0 to the maximum values of 1213.64 mg/l, 538.17 mg/l and 153,600 AU/ml, respectively, at in pH 6.5. Together, the above results provide an economical strategy that is suitable the production of rMP1102 at the industrial level under the control of the GAP promoter in *P. pastoris* for the first time.

## Methods

### Strains, vectors, and reagents

*Escherichia coli* DH5α (Invitrogen, Beijing, China) was used as the host for plasmid maintenance and amplification. The pPICMP1102 plasmid was constructed and saved in our laboratory. The GAP gene was synthesized by Sangon Biotech (Shanghai, China). *P. pastoris* X-33 (Invitrogen, Beijing, China) was used for the expression. *S. aureus* ATCC 25923 was used in the antimicrobial activity assays. Restriction enzymes were purchased from New England Biolabs (NEB, Beijing, China). The kits for plasmid extraction and DNA purification were purchased from Tiangen (Beijing, China). All other chemical reagents were of analytical grade.

### Construction of pGAPMP1102 and screening of the transformants

The pPICMP1102 recombinant plasmid was constructed in previous work [40]. The AOX promoter of pPICMP1102 was replaced with the GAP promoter in this study, its detail including the restriction sites (*Bgl* II and *Xho* I) was shown in Figure 1. The target peptide was inserted in the downstream of the α-factor, which is most widely and successfully used secretion signal and can be self-cleaved by the Kex2 protease in *P. pastoris*, resulting in the native sequence of target protein. The recombinant plasmid was named pGAPMP1102 (GenBank accession number: KP636420) and transformed into *E. coli* DH5α. The positive transformants were screened by colony PCR and DNA sequencing. The correct positive plasmid was linearized with *Avr*II and transformed into *P. pastoris* X-33 by electroporation at 1,200 V charging voltage. The positive transformants were selected on YPDS plates (20 g/l peptone, 10 g/l yeast extract, 20 g/l glucose, 182.2 g/l sorbitol, and 20 g/l agar) containing 100 μg/ml Zeocin. The transformants were further confirmed by colony-PCR.

### Expression of rMP1102 and optimization of cultivation in shaking flasks

The positive transformants were cultured overnight at 29°C and 250 rpm in 50-ml shaking flasks containing 10 ml YPD medium (20 g/l peptone, 10 g/l yeast extract, 20 g/l glucose). A 500-μl overnight culture was inoculated into a 250-ml shaking flask containing 50 ml YPD medium. Glucose (50%) was repeatedly added every 24 h to a final concentration of 0.5-1.0% (v/v) during the 72 h expression period, and the cell supernatants was collected every 24 h. The collected supernatants were centrifuged at 12,000 rpm for 10 min and stored at −20°C.

The colony with the highest expression level was selected and grown in 10 ml YPD medium overnight. This cell culture was further inoculated to an optical density (OD600) of 0.1-0.3 to initiate the cell growth and protein expression in the modified YP media (1% (w/v) yeast extract, 2% (w/v) peptone) supplemented with different carbon sources (i.e., glucose, glycerol, or methanol) at different concentration of 1, 2, 3, 4, and 5% (w/v). The culture supernatants were collected, and total protein levels were measured with a Bradford protein assay kit (Tiangen Biotech, Beijing, China). The yield of rMP1102 was calculated with Quantity One software, Version 4.6.2 (Bio-Rad, USA). The antimicrobial activity was assayed by the method described in the “Antimicrobial activity assays” section.

Based on the results of the carbon source screening, six different media (Table 4) were used to select the most suitable medium for large-scale production. In detail, a single colony of the recombinant strain was grown in 10 ml of YPD medium overnight and inoculated into 50 ml of YPD medium. The cell culture was further inoculated to an optical density (OD600) of 0.1-0.3 to initiate the cell growth and protein expression in 200 ml volumes of different media (Table 4). Glucose (50%) was repeatedly added every 24 h to a final concentration of 4.0% (v/v) during the 96 h expression period, and the cell supernatants were collected every 24 h. The total protein levels, rMP1102 yield and antimicrobial activities were tested. Tricine-SDS-PAGE was used to evaluate the expression levels of the target peptides. In this system, tricine is used instead of glycine in the cathode buffer due to that its stronger negative charge than glycine allows peptide to migrate faster. In addition, more ion movement and less protein movement from high ionic strength improves small proteins to be separated [41].

**Table 4:** **Composition of media used in rMP1102 production assays**

	**Med-1 (Modified FM22)**	**Med-2**	**Med-3 (BSM)**	**Med-4**	**YPD**	**BMGY**
KH_2_PO_4_	42.9	4	—	6	—	—
H_3_PO_4_	—	—	26.7 ml	—	—	—
(NH4)_2_SO_4_	5	4	—	—	—	—
NH_4_H_2_PO_4_	—	—	—	50	—	—
CaSO_4_	0.6		0.93	0.4	—	—
CaCl_2_	—	0.38	—	—	—	—
K_2_SO_4_	14.3	18.2	18.2	20	—	—
MgSO_4_•7H_2_O	11.7	9.4	14.9	15	—	—
KOH	—	—	4.13	1.5	—	—
Citric acid	1.92	—	—	—	—	—
Glucose	40	40	40	40	40	40
Peptone	—	—	—	—	20	20
Yeast extract	—	—	—	—	10	10
PTM1	0.48%	0.48%	0.48%	0.48%	0.48%	0.48%
Reference	[42]	[35]	[43]	[16]	—	—

To further improve the rMP1102 yield, 2% peptone and 1% yeast extract from Oxoid (which are commonly used for research) and from Hongrun Baoshun (HRBS, these were crude and industrial grade) were added to Med-1, which was the medium that resulted in the maximum production of the target peptides. The methods for the cultivation, and total protein level and Tricine-SDS-PAGE assays were those described in the preceding paragraph.

### Fed-batch fermentation at different pHs

The fermentation studies were conducted in 5-l bioreactors (BIOSTAT®B plus, Sartorius Stedim Biotech). A single colony was incubated in YPD medium at 30°C. An overnight culture was inoculated into 200 ml of fresh YPD medium and cultivated at 29°C (250 rpm) to an OD600 of 4–6. Next, 10% (v/v) inoculum was inoculated into a 5-L fermenter containing 2.0 L modified Med-1 medium (42.9 g/L KH_2_PO_4_, 14.3 g/l K_2_SO_4_, 11.7 g/l MgSO_4_ 7H_2_O, 5 g/l (NH) _2_SO_4_, 1.92 g/l citric acid anhydrous, 0.6 g/l CaSO_4_, 20.0 g/L yeast extract, 40.0 g/L peptone, 40.0 g/L glucose). The pH value was set to 6.0 at the glucose batch phase and changed to 5.0, 5.5, 6.0, 6.5, or 7.0 in the glucose fed-batch phase using NH_4_OH and H_3_PO_4_. The glucose feeding rate was 6 mL/L/h during the fed-batch culture phase. The dissolved oxygen (DO) level was maintained between 25 and 40% by adjusting the agitation, aeration, and rates. Foaming was controlled via the addition of antifoam. Cultivation broth samples were taken every 12 h for cell wet weight, extracellular protein level and antimicrobial activity analyses after centrifugation at 12,000 rpm for 10 min. The total protein levels, rMP1102 yield and antimicrobial activities were tested.

### Purification and identification of rMP1102

The fermentation supernatant was dialyzed using a Sephadex G-25 column and lyophilized. The dried powder was dissolved in 20 mM of sodium phosphate buffer at a pH of 6.7 and loaded onto a HiTrap SP FF cation exchange chromatography (cIEX) column (length, 25 mm; internal diameter, 7 mm; GE Healthcare). First, 20-mM sodium phosphate buffer and 140-mM NaCl at a pH of 6.7 were used for elution, the concentration of NaCl was then increased to 600 mM, and the eluent of the corresponding elution peak was collected. Protein elution was monitored by measuring the UV absorbance at 215 and 280 nm.

The purified rMP1102 was analyzed by tricine-SDS-PAGE and confirmed by MALDI-TOF MS at the Laboratory of Proteomics, Institute of Biophysics, the Chinese Academy of Sciences according to previously reported methods [24]. The purity of the target peptide was calculated with Quantity One software, version 4.6.2 (Bio-Rad, USA).

### Antimicrobial activity assays

The tested strain of *S. aureus* ATCC 25923 was grown at 37°C to an OD600 of 0.4 in Mueller–Hinton (MH) medium (5 g/l beef infusion solids, 17.5 g/l casein hydrolysate, 1.5 g/l starch, and 3% serum, pH7.4). The cell suspension was inoculated into preheated MHA (5 g/l beef infusion solids, 17.5 g/l casein hydrolysate, 1.5 g/l starch, 20 g/l agar, 3% serum, pH 7.4). The medium was rapidly mixed and poured into a petri dish. Sterile oxford cups were placed on the surface of solidified the medium, and each cup was filled with 50-μl samples and incubated for 16–18 h at 37°C [44].

A titer assay was used to quantify the antimicrobial activity, which is expressed as arbitrary units (AU/ml). One arbitrary unit (AU) was defined as the reciprocal of the highest dilution that exhibited a clear zone of inhibition with the indicator strain. When a clear inhibition zone was followed by a turbid zone, the critical dilution was taken as the average of the final two dilutions [42].

### Statistical analyses

All statistical analyses were performed using SPSS version 22.0. One-way analysis of variance (ANOVA) was used to determine the significance of the differences between groups. Differences were considered significant at P < 0.05.
